# Profiles of *Bacillus* spp. Isolated from the Rhizosphere of *Suaeda glauca* and Their Potential to Promote Plant Growth and Suppress Fungal Phytopathogens

**DOI:** 10.4014/jmb.2105.05010

**Published:** 2021-07-15

**Authors:** Ping Lu, Ke Jiang, Ya-Qiao Hao, Wan-Ying Chu, Yu-Dong Xu, Jia-Yao Yang, Jia-Le Chen, Guo-Hong Zeng, Zhou-Hang Gu, Hong-Xin Zhao

**Affiliations:** 1 Zhejiang Province Key Laboratory of Plant Secondary Metabolism and Regulation, College of Life Sciences and Medicine, Zhejiang Sci-Tech University, Hangzhou 310018, P.R. China; 2 Experimental Teaching Center, College of Life Science, Shenyang Normal University, Shenyang 110034, P.R. China

**Keywords:** *Bacillus* spp., *Suaeda glauca*, plant biocontrol, plant growth promotion, *Arabidopsis thaliana*

## Abstract

Members of the genus *Bacillus* are known to play an important role in promoting plant growth and protecting plants against phytopathogenic microorganisms. In this study, 21 isolates of *Bacillus* spp. were obtained from the root micro-ecosystem of *Suaeda glauca*. Analysis of the 16S rRNA genes indicated that the isolates belong to the species *Bacillus amyloliquefaciens*, *Bacillus velezensis*, *Bacillus subtilis*, *Bacillus pumilus*, *Bacillus aryabhattai* and *Brevibacterium frigoritolerans*. One of the interesting findings of this study is that the four strains B1, B5, B16 and B21 are dominant in rhizosphere soil. Based on *gyrA*, *gyrB*, and *rpoB* gene analyses, B1, B5, and B21 were identified as *B. amyloliquefaciens* and B16 was identified as *B. velezensis*. Estimation of antifungal activity showed that the isolate B1 had a significant inhibitory effect on *Fusarium verticillioides*, B5 and B16 on *Colletotrichum capsici* (syd.) Butl, and B21 on *Rhizoctonia cerealis* van der Hoeven. The four strains grew well in medium with 1-10% NaCl, a pH value of 5-8, and promoted the growth of *Arabidopsis thaliana*. Our results indicate that these strains may be promising agents for the biocontrol and promotion of plant growth and further study of the relevant bacteria will provide a useful reference for the development of microbial resources.

## Introduction

*Bacillus* species are a group of gram-positive bacteria widely existing in nature [1]. They can produce notable and useful secondary metabolites and are found in diverse environments such as water, soil, vegetation, and even the gastrointestinal tracts of various insects and mammals [2]. This ability to survive and grow in such varied ecosystems is based on the production of endospores that are highly resistant to unfavorable environmental conditions, in addition to their diverse physiological properties and growth requirements [3, 4]. When *Bacillus* species are applied in agriculture, they can bring all kinds of benefits to plants, including strengthening resistance to disease caused by plant phytopathogenic fungi and bacteria, or insects and nematodes [5]. At the same time, they can improve tolerance to stress while promoting plant growth and being eco-friendly [5, 6]. Many species of *Bacillus* have been found to secrete gibberellins, plant hormones such as Indole-3-acetic acid (IAA), and some nutrient-solubilizing enzymes that promote the rapid growth of plants [7]. Some physiological changes when plants were inoculated with *Bacillus* spp. in a stress environment slow down plant senescence [7]. Bacilli also secrete secondary metabolites such as antibiotics, siderophores, and cell wall hydrolases, which contribute to their antagonistic effects against pathogens [5, 8]. In addition, *Bacillus* spp. can elicit systemic resistance to improve plant resistance against subsequent pathogen attack [8]. It is well known that the roots of plants in the soil are colonized by a plethora of different bacteria and fungi which are largely affected by the physical and chemical properties of the soil and plant genotypes [9, 10]. However, few reports have investigated the rhizospheric and rhizophilous bacterial microflora in the root micro-ecosystems of halophytes.

*Suaeda glauca* is a succulent obligate halophyte from the family Chenopodiaceae, and one of the few plant species that can live in highly saline and alkaline soils [11]. It is a significant pioneer species in coastal wetlands of East Asia, and is widely distributed in coastal areas of China [12]. What’s more, it is not only an indicator of saline soils but also an ideal plant variety for improving the arability of saline-alkali soils and phytoremediation [11, 13]. The introduction of halophytes such as *S. glauca* is beneficial for increasing land utilization rates, enhancing the value of saline and solonetzic soils, and economical remediation of mine tailings [12]. In addition to efficiently reducing the salt content of topsoil in which it is cultivated, *S. glauca* can increase the content of organic matter content as well as nitrogen, phosphorus, and potassium levels in the soil, which generates economic, ecological and social benefits [14, 15]. These extremely advantageous characteristics make this plant a mighty candidate for saline alkali agriculture and an important source of salt and drought tolerant bacteria, which is why we chose this plant in our research.

In the present study, we set out to explore the diversity of *Bacillus* species and find novel functional strains. We isolated typical *Bacillus* spp. from the root micro-ecosystem of *S. glauca* Bunge, which develops naturally in saline and solonetzic soils with high tolerance of the relatively harsh environmental conditions of Red Beach at Panjin in Liaoning Province (40°41’-41°27’N, 121°31’-122°28’E), which is one of the best preserved wetlands in China. The antimicrobial activity and plant growth-promoting effects of selected strains were characterized, and four selected strains were analyzed in more detail. This study provides a reference for exploiting novel microbial resources and as well as for future investigations of the relationship between the dominant plants and associated microorganisms in saline-alkali soils.

## Materials and Methods

### Reagents and Culture Media

The Qubit 2.0 DNA Kit used for genomic DNA isolation was purchased from NEB (USA). The Taq DNA Polymerase and High-Fidelity DNA Polymerase used for the amplification of 16S rRNA genes were purchased from Thermo Fisher Scientific (USA). The DNA gel extraction kit was purchased from GenScript (USA). Unless specifically noted, all other chemicals were obtained from Sigma-Aldrich (USA).

Luria-Bertani (LB) broth containing 10 g/l tryptone, 5 g/l yeast extract, and 10 g/l NaCl was used for the general cultivation of bacteria or bacterial isolates. *Bacillus* spp. were grown in LB medium at 30 or 37°C. Potato dextrose agar (PDA) containing 200 g peeled potatoes, 20 g glucose, 15 g agar, and 1,000 ml water was used for culturing plant pathogenic fungi. Murashige and Skoog (MS) medium (Sigma-Aldrich) was used as a plant growth medium [16].

### Sample Collection and Isolation of *Bacillus* spp.

Eight *S. glauca* Bunge and soil collection sites and ten samples of soil were selected at Red Beach (40°41’-41°27’N, 121°31’-122°28’E). Samples of *S. glauca* Bunge with rhizosphere soil samples were collected from different soil depths (0-10 cm) and stored at 4°C.

*Bacillus* strains were isolated from ten samples by serial dilution in conjunction with the agar plate method [17]. Soil samples weighing 5 g were ground and mixed with 9 ml of autoclaved distilled water, shaken thoroughly, and then kept at 70°C for 1 h. A 1 ml mixture of this solution was diluted with sterile distilled water at a ratio of 1:10. In addition, each soil sample was serially diluted 10^-3^ to 10^-8^. Then, 0.1 ml of each dilution was spread on two LB agar plates, one of which was incubated at 30°C and the other at 37°C, for 7 days. Every single colony was picked and sub-cultured on LB agar medium for purification and further studies of colony morphology. Pure bacterial culture suspensions were then grown in LB medium and optical density of cultures at 600 nm (OD_600_) up to 1.5, and stored as 20% glycerol stock at -80°C.

### 16S rRNA Sequencing and Phylogenetic Analysis of *Bacillus* spp.

Genomic DNA of the bacteria isolated from the root micro-ecosystem of *S. glauca* Bunge was extracted using the bacterial genomic DNA FastPrep Extraction Kit (Sangon Biotech, China). DNA was quantified on a Nanodrop ND100 spectrophotometer (Thermo Fisher Scientific) and stored at -20°C.

The 16S rRNA genes from rhizobacterial isolates were amplified using the bacterial universal primers 27F (5′-AGTTTGATCMTGGCTCAG-3′) and 1492R (5′- GGTTAC CTTGTTACGACTT-3′). PCR was performed in a T1 cycler (Biometra, Germany) in 50-μl reaction systems containing 0.5 μM of each primer, 25 μl 2×Taq Master Mix (Dye Plus) (Vazyme, China), 1 μl of genomic DNA and sterile distilled water (up to 50 μl). Thermal cycling was carried out with a denaturation step at 94°C for 3 min, followed by 30 cycles of 45 s denaturation at 94°C, 45 s annealing at 56°C and 90 s elongation at 72°C, with a final elongation step at 72°C for 5 min, then cooled to 4°C. The PCR products were further sequenced and verified by Sangon Biotech (China) after purifying with a QIAquick-PCR purification column (Qiagen, Germany). A sequence similarity search was conducted using GenBank BLAST. The phylogenetic tree was constructed using MEGA7 software with bootstrap analysis using 1,000 replications to assess the relative stability of the branches. All 16S rRNA gene sequences from the isolates were submitted to the NCBI GenBank Database under an accession number.

### Cloning and Partial Sequence Analysis of the *gyrA*, *gyrB* and *rpoB* Housekeeping Genes

The partial sequences of the three housekeeping genes *gyrA*, *gyrB*, and *rpoB* were amplified using the specific primers *gyrA* F (5′-CAGTCAGGAAATGCGTACGTCC TT-3′): *gyrA* R (5′-CAAGGTAATGCTCCAGGCATTGCT-3′), *gyrB* F (5′-TTATCTACGACCTTAGA CG-3′): *gyrB* R (5′-TAAATTGAAGTCTTCTCCG-3′) [18], and *rpoB* F (AGGTCAACTAGT TCAGTATGGAC): *rpoB* R (5′-AAGAACCA TAACCGGCAACTT-3′), respectively [19]. The 50 μl PCR reaction system contained 5 μl of the 10× PCR buffer, 2 μl dNTPs, 1 μl Pfu DNA polymerase (Takara Bio Inc., China), 3 μl genomic DNA template (25 ng/μl DNA), 2 μl of each of the primers (0.1 μM) and 35 μl of the deionized distilled water (DDW). The temperature program included a denaturation step at 94°C for 5 min, followed by 30 cycles of 95°C for 1min, annealing at 50~63°C for 30 s, and elongation at 72°C for 1 min, followed by a final elongation cycle at 72°C for 5 min, after which the PCR product was kept at 4°C. For Sanger sequencing, 20 μl of the PCR product purified using the DNA gel extraction kit (Qiagen) was sent to Sangon Biotech (China). Results of sequencing were analyzed using Chromas V 1.45, DNAMAN 8.0, MEGA7 and Blast software at the NCBI site. The partial sequences of the tree housekeeping genes from the isolates were submitted to the NCBI GenBank Database under an accession number (Fig. 3).

### Screening for Antifungal Bacteria and Testing of Antifungal Activity

Twelve plant-pathogenic fungi (including *Fusarium oxysporum*, *Fusarium graminearum* Sehw, *Rhizoctonia cerealis* van der Hoeven, *Gaeumannomyces graminis* (sacc.), *Botrytis cinereal* Pers., *Botryosphaeria dothidea*, *Colletotrichum gloeosporioides* Penz, *Fusarium oxysporum* f. sp. *niveum*, *Colletotrichum capsici* (syd.) Butl., *Fusarium verticillioides*, *F. oxysporum* f. sp. *vasinfectum* (Atk) and *F. oxysporum* f. sp. *lilii* (for more details see Table S1), all of which were originated from plants including agricultural crops, fruits and vegetables, were provided by the Institute of Plant and Environment Protection (IPEP) of Beijing Academy of Agriculture and Forestry Sciences (BAAFS) and exposed to the isolated bacteria to assess their antifungal spectrum. To measure the antifungal activity of the bacteria, a mycelial disk (5 mm in diameter) of each plant-pathogenic fungus from a 5-day-old culture was placed at the edge of a PDA medium plate, 25 mm away from each streaked bacterial inoculum (10^6^ CFU/ml), which was placed in the middle of the plate. Then, the plate was incubated at 25°C for 5-8 days. Inhibition of mycelial growth of each fungus was then measured, and the inhibitory effect of the antagonistic bacteria was assessed by the radial growth inhibition percentage.

### Tolerance of Isolates to Salinity and Alkalinity

The antagonistic bacteria screened in the antifungal activity test were then further characterized for their ability to tolerate salinity and alkalinity. The NaCl concentration in the LB broth was varied from 0.1% to 15%, and the pH value was adjusted from 3 to 10 with 1 mol/l sodium hydroxide (NaOH) or 1 mol/l hydrochloric acid (HCl) for the alkalinity test. The seed cultures of the antagonistic strains were used to inoculate 250-ml flasks containing 30 ml of LB medium and grown at 30°C and 150 × *g* for 20 h. Cell proliferation was monitored by measuring the OD_600_.

### Ability of the Isolates to Promote the Growth of *Arabidopsis thaliana*

Seeds of *A. thaliana* ecotype Columbia (Col-0) were surface-sterilized by soaking in 75% ethanol for 2 min, followed by soaking in 1% sodium hypochlorite (NaOCl) for 20 min. The seeds were thoroughly washed three times with sterile distilled water and then germinated in Petri dishes with half-strength Murashige and Skoog (MS) medium containing 0.8% agar and 1.5% sucrose, and the pH was adjusted to 5.7 [16]. The seeds were then vernalized for 2 days at 4°C in darkness. The resulting seedlings were placed in growth cabinets and subjected to a 12-h light/dark cycle under 40W fluorescent lights at a constant temperature of 22°C and relative humidity of 50-60%. Germinated seedlings were transferred to plates after 2 days to test the ability of the bacterial strains to promote the growth of *A. thaliana* [16].

The bacterial strains were cultured overnight at 30°C in LB medium, and then diluted with sterile distilled water to yield 10^9^ CFU/ml based on the OD_600_ and the number of colonies counted on plates for the various serial dilutions. Plastic Petri dishes (100 × 15 mm) with a central partition were filled with Murashige and Skoog solid medium, and 2-day-old germinated *A. thaliana* seedlings (9 seedlings per plate) were transferred to one side of each plate. The plants were inoculated with 10 μl bacterial strain solution (10^9^ CFU/ml) or sterile distilled water (as control) at the center of the other side of the plate that did not contain the seedling. After 2 weeks of incubation, the total number of leaves and the leaf and fresh stem weights were measured to evaluate the growth-promoting effect of the bacteria [16]. The assay was repeated three times each with nine biological replicates (*n* =3×9=27) for each treatment.

### Statistical Analysis

The significance of differences was assessed using analysis of variance (ANOVA) in SPSS20.0 software (IBM Corp., USA). The antibacterial effect of four *Bacillus* sp. strains on 12 fungal pathogens was determined by the following formula: IR = (R1−R2)/R1 × 100%, where R1 is the radial growth of the plant-pathogenic fungus on the control plate, and R2 is the radial growth of the plant pathogenic fungus interacting with the antagonistic strain. The effect of the treatment with growth promotion of *A. thaliana* was determined based on the magnitude of the F value (*p* = 0.05). When the ANOVA was significant (*p* ≤ 0.05), means were separated with Duncan’s test.

## Results

### Isolation and Screening of *Bacillus* spp. from the Root Micro-Ecosystem of *S. glauca* Bunge

Eight soil collection sites encompassing diverse habitats such as offshore, riverway, tributary, swale, etc., were selected at Red Beach, and ten soil samples from the rhizosphere of *S. glauca* Bunge (labeled as Sg-01 through Sg-10) were collected and refrigerated at 4°C. A total of 103 strains were isolated as described in Materials and Methods. The isolates were further identified based on their morphological characteristics such as size, color, colony edge shape, and viscosity, and also according to the results of Gram staining and microscopic observation. Based on obviously different morphological characteristics, 21 isolates were suspected to be different strains (see Table S2 in the supplemental material) and chosen for further identification by 16S rRNA gene sequencing.

**Fig. 1:**
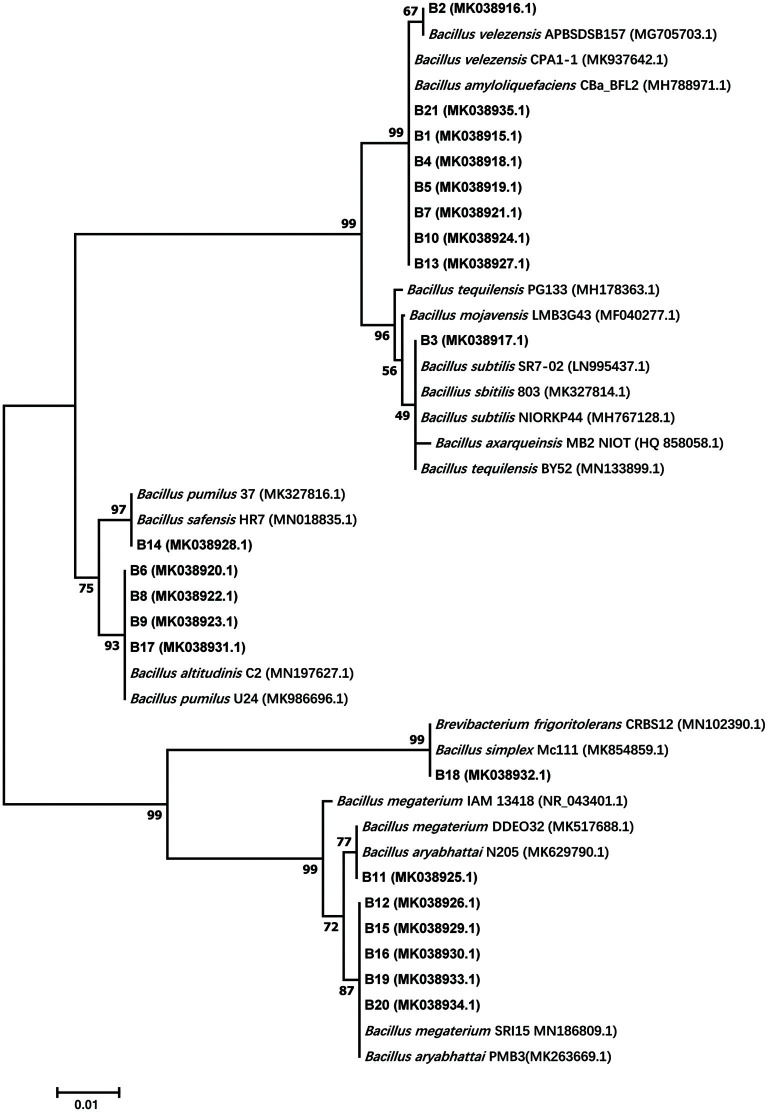
Phylogenetic tree based on the 16S rDNA sequences of *Bacillus* spp. strains isolated from the rhizosphere of *S. glauca* constructed using the neighbor-joining method.

The analysis of 16S rRNA sequences indicated that the 21 isolates, which were collected from the root micro-ecosystem of *S. glauca* Bunge, could be categorized into five clusters (Fig. 1). However, the isolates belonged to only two genera, *Brevibacterium* and *Bacillus*. Among the isolates, only a single strain of *Brevibacterium* was obtained, and was identified as *Brevibacterium frigoritolerans*, while among the *Bacillus* species, eight strains were identified as *Bacillus velezensis*, one was *Bacillus subtilis*, five were *Bacillus pumilus*, and six were determined as *Bacillus aryabhattai*.

### Screening and Analysis of the Antifungal Activity of the Isolated *Bacillus* Strains

**Fig. 2:**
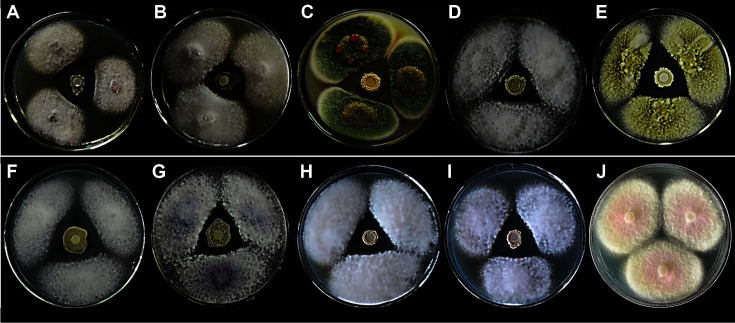
Antifungal effects of the isolated *Bacillus* spp. strains against different plant-pathogenic fungi (**A**: B1 against *F. oxysporum*; **B**: B1 against *B. cinerea* Pers.; **C**: B1 against *G. graminis* (sacc.); **D**: B5 against *R. cerealis* van; **E**: B5 against *C. capsici* (syd.) Butl.; **F**: B16 against *F. oxysporum* f. sp. lili; **G**: B16 against *F. oxysporum*; **H**: B21 against *B. dothidea*; **I**: B21 against *B. cinerea*l Pers; **J**: *F. graminearum* Sehw against *F. graminearum* Sehw as one of the controls).

The 21 isolated bacterial strains were tested for their ability to inhibit or prevent mycelial growth of fungal plant pathogens, and four *Bacillus* sp. strains labeled as B1, B5, B16 and B21 showed apparent antifungal activity, especially against *C. capsici* (syd.) Butl., *F. verticillioides*, *R. cerealis* van, *G. graminis* (sacc.), *B. cinerea* Pers. and *B. dothidea* (Fig. 2 and Table 1).

Table 1 lists the results of the antagonistic activity test for each isolate. The inhibition rates of the four *Bacillus* sp. strains were monitored by measuring the width of the inhibition zone (see Table S3 in the supplemental material). Strain B1 was more effective against *F. oxysporum* f. sp. *vesinfectum* (Atk.) and *F. oxysporum*, with 67.69% and 67.41% antimicrobial rate, respectively. Strain B5 was the most effective strain against *C. capsica* (syd.) Butl., with 72.95% antimicrobial rate, followed by 66.17% and 65.08% antimicrobial rate against *F. verticillioides* and *F. oxysporum* f. sp. *lilii*. Strain B16 was the most effective bacterium against *C. capsica* (syd.) Butl. (74.56%), followed by 70.22% and 67.41% antimicrobial rate against *B. dothidea* and *F. oxysporum* f. sp. *niveum*, whereas strain B21 was the most effective against *R. cerealis* van up to 73.52%, and close to *B. dothidea* up to 71.66%. These results demonstrated that all four *Bacillus* strains exhibited broad-spectrum antifungal activity.

### Analysis of *gyrA*, *gyrB*, and *rpoB* Housekeeping Genes, and 16S rRNA Gene from B1, B5, B16, and B21

**Fig. 3:**
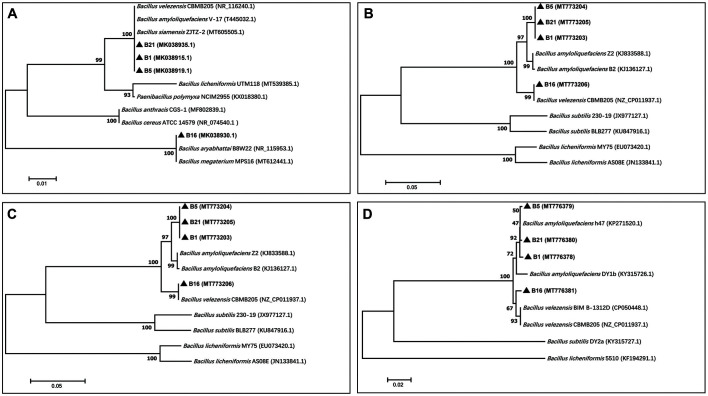
Phylogenetic tree of the antifungal *Bacillus* strains B1, B5, B16, and B21 generated using the neighbor-joining method based on the 16S rDNA gene sequences (**A**), and the *rpoB* (**B**), *gyrA* (**C**), and *gyrB* gene sequences (**D**).

Based on the inhibition of the mycelial growth of fungal plant pathogens, B1, B5, B16, and B21 appeared to have distinct antifungal activity. However, the 16S rRNA analysis showed that B1, B5, and B21 were strains of *B. velezensis*, with sequence identities from 99 to 100%. The 16S rRNA gene sequence sometimes has a limited ability to reliably distinguish taxa at the species level due to the conserved nature of the gene [19]. In order to further phylogenetically analyze B1, B5, B16, and B21, the housekeeping genes *gyrA*, *gyrB*, and *rpoB*, which respectively code for major subunits of the DNA Gyrase and RNA polymerase, were also employed for identification [18, 19]. Phylogenetic trees were constructed using the 16S rRNA gene sequences (Fig. 3A), the *rpoB* gene sequence for B1, B5, B16, and B21 (Fig. 3B), the *gyrA* gene sequence (Fig. 3C), and the *gyrB* gene sequence (Fig. 3D). The analysis of the 16S rRNA genes of B1, B5, B16, and B21 analysis showed that B1, B5, and B21 clustered with *B. velezensis*, *B. amyloliquefaciens* or *B. siamensis* while B16 clustered with *B. aryabhattai* or *B. megaterium* (Fig. 3A). The sequence analysis of the housekeeping gene *rpoB*, which encodes one of the subunits of RNA polymerase, differentiated the isolates B1, B5, B21, and B16. The analysis of *rpoB* revealed that B1, B5, and B21 showed high similarity (Fig. 3B), while B16 was more distant. Based on *gyrA* gene sequence analysis, B1, B5, B21 were identified as *B. velezensis*, and B16 was identified as *B. amyloliquefaciens* (Fig. 3C). Based on *gyrB* gene sequence analysis, B1, B5, B21 were identified as *B. amyloliquefaciens* and B16 was identified as *B. velezensis* (Fig. 3D). The nucleotide and amino acid changes in *gyrA*, *gyrB*, and *rpoB* of B1, B5, B16, and B21 compared with the reference strain *B. velezensis* strain CBMB205 (NZ_CP011937.1), and the frequency of nucleotide and amino acid substitutions among the different groups are listed in Table 2. The *gyrA* sequence of B16 was identical to that of *B. velezensis*, while G (457) and T (573) of *gyrA* in the other strains were changed into T and C, respectively, whereas Ala (153) and Val (158) were changed into Ser and Ala accordingly. T (324) of *rpoB* in the four strains was changed into C and G (276) of B1, B5, and B21 were changed into A, but all of their amino acid sequences showed no differences. A (548) of *gyrB* in B1, B5, and B21 was changed into T, C (494) in B1, T (533) in B5, G (460) in B21, and G (884) in B16 were changed into T, G, A, and A, respectively. Accordingly, Glu (183) in B1, B5, and B21 was changed into Val, while Thr (163) in B1, Phe (178) in B5, Ala (154) in B21 and Arg (195) in B16 were respectively changed into Ile, Cys, Thr, and His. The comparison results of the three housekeeping genes *gyrA*, *gyrB*, and *rpoB* showed that B1, B5, and B21 could belong to different strains of the same species called *B. amyloliquefaciens*.

### Salinity and Alkalinity Resistance of the Antifungal *Bacillus* Strains

**Fig. 4:**
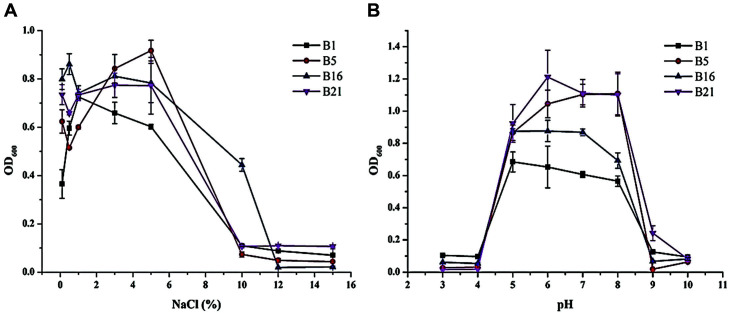
Resistance to salinity and alkalinity of the four antifungal *Bacillus* strains.

The effects of salinity and alkalinity on the proliferation of the strains B1, B5, B16, and B21, which exhibited broad-spectrum antifungal activities, were also assessed (Fig. 4). Salt tolerance differed substantially among the strains. The salt concentration that could be tolerated by the strains ranged from 0.1% to 10% (Fig. 4A). All strains grew well at 1% salt concentration (10 g/l NaCl; *i.e.*, normal LB medium), with a growth potential of up to 10%. The OD_600_ of B5 reached its maximum of 0.9 when the concentration of NaCl was 5%. However, only B16 could grow in medium with 10% NaCl. The relative salt tolerance of the strains within the NaCl range 1-10% was in the order B5 > B16 > B21 > B1. All strains stopped growing when exposed to salt concentrations above 12%.

An acid-alkali tolerance test was carried out in LB media with different pH values (3 to 10). The four *Bacillus* strains all grew normally in the pH range of 5-8 (Fig. 4B), but their optimal pH values were quite different as the strains B1 and B16 grew best at pH 5-7, while B5 and B21 showed the best growth at pH 6-8. None of the strains grew well at pH ≤ 4 or > 8.

### Ability of the Four *Bacillus* sp. Strains to Promote the Growth of *A. thaliana*

**Fig. 5:**
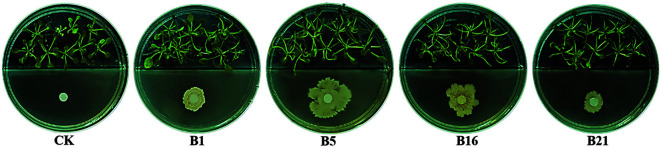
Plant growth promotion analysis of the four *Bacillus* sp. strains on *A. thaliana* compared with water treatment (control).

To assess the growth-promoting effect of the four antimicrobial strains, *A. thaliana* was cultivated with B1, B2, B16, B21 and sterile water (as control) in solid medium on the same plate (Fig. 5) (*n* = 27). Compared with the control group, each strain could promote the growth of *A. thaliana*, albeit to different extents. The growth-promoting effect was assessed based on the increase of the number of leaves and the fresh weight of the combined stems and leaves (Table 3). Strains B1, B5, and B21 significantly promoted the growth of leaves, while strains B1, B16, and B21 were relatively more effective in promoting the increase of leaf-stem fresh weight.

## Discussion

Plant roots growing in special environments such as wetlands or highly alkaline soil can produce a broad variety of metabolites to adapt to the environment and attract or select special microorganisms in the rhizosphere [20]. A variety of microorganisms form a complex micro-ecosystem and establish a dynamic balance with plants [21]. However, there are few studies on the diversity and distribution of micro-ecosystem and endophytic bacteria associated with plants and their potential functions [21, 22]. Previously, we analyzed the microbial colony structure of the *S. glauca* Bunge root micro-ecosystem by high-throughput sequencing [23]. The analysis yielded a total of 143,041 effective sequences, 1,346 OTUs, 27 phyla, 60 classes, 104 orders, 193 families, 355 genera, and 408 species [23]. The abundance of microbial species inhabiting the roots of *S. glauca* may provide clues for understanding the salt and alkali tolerance traits of *S. glauca* [24]. Thus, the results in this investigation may provide further detailed information for the composition and diversity of the rhizosphere bacterial microbiome of the halophyte *S. glauca* Bunge. In order to expand our understanding of the diversity of culturable salt-tolerant rhizospheric bacteria from the roots of halophyte *S. glauca* Bunge, we isolated and identified different species of rhizospheric bacteria from 10 sampling sites. However, it is worth mentioning that 103 strains were isolated from the root micro-ecosystem of *S. glauca* Bunge. According to the obviously different morphological characteristics, 21 isolates were suspected to be different strains. Based on the analysis of 16S rRNA sequences, the isolates belonged to only two genera, *Brevibacterium* and *Bacillus*. *B. velezensis*, *B. subtilis*, *B. pumilus*, *B. aryabhattai* and *B. frigoritolerans* were found among the identified isolates. It was shown that the *Bacillus* spp. is the dominant phyla in *S. glauca*. Similar phyla were presented by Yamamoto *et al*. [25] who studied the diversity of endophytic and rhizospheric bacteria of the halophytes *S. europaea* and *Glaux maritima*. What’s more, Bouizgarne [26] reported that *Bacillus* spp. could mainly colonize in the rhizosphere of plants. Generally, *Bacillus* spp. may play significant roles in the life of *S. glauca* and other halophytes [27-29], as bacteria could produce certain antifungal and antibacterial metabolites [30-32] including the notable proteins iturin and chitinase [5].

Rhizosphere bacteria are well known inhabitants of living plant systems and play an important role in maintaining plant growth and health. In this examination, four *Bacillus* sp. strains labeled as B1, B5, B16, and B21 showed apparent antifungal activity, which may indicate their high antifungal ability under saline conditions. In another investigation, fresh fruits such as strawberry and golden delicious were infected with *B. cinerea* Pers., and fought against with *Bacillus* sp. (B16) at room temperature for more than 12 days. The results showed that *Bacillus* sp. (B16) also has good resistance to plant pathogenic bacteria on real fruits (data not shown). Based on the data obtained from the literature, *Bacillus* species obtained from soil or aerial parts of plants have been proven to be superb producers of antibacterial compounds and have the potential to control fungi and phytopathogenic bacteria [33, 34]. Kupper *et al*. [35] found that most *Bacillus* isolates produce volatile metabolic compounds that inhibit the development of pathogens and the production of two antibiotics (iturin and surfactant) can be detected in *B. subtilis* ACB-83. Similar results were found by Asari *et al*. [36] who used *B. amyloliquefaciens* strains to control pathogens and showed that the isolates can effectively produce volatile organic compounds with antibacterial effects in vitro. And Leelasuphakul *et al*. [37] found that volatile organic compounds (alcohols) produced by *Bacillus* can inhibit the growth of fungi. Furthermore, based on the genome sequence analysis of *B. velezensis* C4341 isolated from saline soil samples, Zhu *et al*. [38] presented that genes related to biofilm formation, iron acquisition, colonization, and synthesis of volatile organic compounds were found in the genome of *B. velezensis*. These genes play an important role in the process of biological control. Notably, it seems that four *Bacillus* sp. strains not only have high antifungal ability but also other abilities such as promoting the growth of plants. To assess the growth-promoting effect of the four antimicrobial strains, *A. thaliana* was cultivated with B1, B5, B16, B21 and sterile water (as control) in solid medium on the same plate. All of the *A. thaliana* showed a significant enhancement in plant growth compared with control seedlings (Fig. 5). Recently, several *Bacillus* spp. including B1, B5, B16, and B21, were also investigated for their ability to promote the growth of *Nicotiana benthamiana* (data not shown). The results indicated that the growth characteristics of *N. benthamiana* were similar to those of *A. thaliana* Columbia (Col-0). These findings were also to a report by Kushwaha, who found various species of endophytic *Bacillus* in pearl millet with excellent growth promotion and biocontrol activities [39]. Therefore, it has been indicated that B1, B5, B16, and B21 have potential applications in agriculture and biotechnology. Moreover, many *Bacillus* isolates can fix nitrogen and also be used for biocontrol of the sugarcane pathogens *Ceratocystis paradoxa* and *Sporisorium scitamineum* [40]. Nitrogen-fixing species can be used as a fertilizer, in addition to their biocontrol effects [40, 41]. Hence, diverse species of the genus *Bacillus* are valuable bioresources for agro-biotechnological applications due to their plant growth-promoting and biological control effects [6], which was consistent with our findings.

Salinity and alkalinity can greatly reduce the growth and productivity of sensitive plants. The effects of salinity and alkalinity on the proliferation of the strains B1, B5, B16, and B21, were also assessed in this examination. However, these strains showed considerable variation in salt and alkaline tolerance. The relative salt tolerance of the strains within the NaCl range 1-10% was in the order B5 > B16 > B21 > B1. Besides, the four *Bacillus* strains all grew normally in the pH range of 5-8, but their optimal pH values were quite different as the strains B1 and B16 grew best at pH 5-7 while B5 and B21 showed the best growth at pH 6-8. This may indicate the high diversity of *Bacillus* spp. in saline environments as well as their adaptation to the plant. However, soil salinity can affect rhizosphere bacterial diversity in normal circumstances and Szymańska reported that the number of endophytic bacteria and rhizosphere bacteria in halophyte *S. europaea* is small under extreme salinity conditions [42]. This may give us some hints about the high salt tolerance of *Bacillus* spp. Although only B16 could grow in medium with 10% NaCl, all strains grew well at 1% salt concentration, and the growth potential could reach 10%. In short, the present results indicated that the diversity and function of *Bacillus* spp. might play an important role in the root micro-ecosystem in *S. glauca*.

## Supplemental Materials

Supplementary data for this paper are available on-line only at http://jmb.or.kr.

## Figures and Tables

**Table 1:** Inhibition rates of the four *Bacillus* sp. strains against 12 fungal pathogens.

The tested strains	Inhibition rate (%)			

	B1	B5	B16	B21
*F. oxysporum*	39.52	25.54	24.73	13.71
*F. graminearum* Sehw	61.51	59.83	62.54	39.23
*R. cerealis* van	47.44	60.35	48.68	73.52
*G. graminis* (sacc.)	26.35	37.04	23.08	8.55
*B. cinereal* Pers.	56.13	43.14	52.51	43.79
*B. dothidea*	58.86	61.40	70.22	71.66
*C. gloeosporioides* Penz	37.61	25.86	20.98	10.34
*F. oxysporum* f. Sp.*niveum*	45.75	66.17	67.41	68.15
*C. capsici* (syd.) Butl.	60.46	72.95	74.56	69.74
*F. verticillioides*	27.02	24.48	15.95	5.31
*F. oxysporum* f. sp. *lilii*	67.41	65.08	67.20	66.67
*F. oxysporum* f. sp. *vesinfectum* (Atk)	67.69	42.31	51.28	48.08

**Table 2:** Classification of BI, B5, B16, and B21 based on distribution of *gyrA*, *gyrB*, and *rpoB* genes nucleotide and amino acid changes.

Gene (5’partial)	Nucleotide change				Amino acid change			

	B1	B5	B21	B16	B1	B5	B21	B16
*16S rDNA*	-	-	-	-	-	-	-	-
*gyrA*	G(457)→T	G(457)→T	G(457)→T	-	Ala(153)→Ser	Ala(153)→Ser	Ala(153)→Ser	-
	T(573)→C	T(573)→C	T(573)→C	-	Val(158)→Ala	Val(158) →Ala	Val(158)→Ala	-
*gyrB*	C(494)→T	T(533)→G	G(460)→A	G(884)→A	Thr(163)→Ile	Phe(178)→Cys	Ala(154)→Thr	Arg(195)→His
	A(548)→T	A(548)→T	A(548)→T	-	Glu(183)→Val	Glu(183)→Val	Glu(183)→Val	-
*rpoB*	G(276)→A	G(276)→A	G(276)→A	T(324)→C	-	-	-	-
	T(324)→C	T(324)→C	T(324)→C	-	-	-	-	-

The *gyrA*, *gyrB*, and *rpoB* from *B. velezensis* CBMB205 (NZ_CP011937.1) as reference, when phylogenetically analyzing B1, B5, B16, and B21 by three housekeeping genes cloned from B1, B5, B16, and B21. The 16S rDNA gene from *B. velezensis* CBMB205 and *B. aryabhattai* B8W22 as reference, when phylogenetically analyzing B1, B5 and B21, and B16 by 16s rDNA genes cloned from B1, B5, B21, and B16, respectively. “-” means no change.

**Table 3:** Abilities of four *Bacillus* sp. strains to promote growth of *A. thaliana* (*n* = 27).

Treatment	Number of leaves	Leaf and stem fresh weight (mg)
CK	8.88 ± 0.99^c^	25.39 ± 8.28^c^
B1	10.38 ± 0.74^ab^	30.79 ± 6.50^bc^
B5	11.25 ± 1.04^a^	38.38 ± 8.62^ab^
B16	9.75 ± 0.71^bc^	40.89 ± 3.72^a^
B21	11.00 ± 1.07^a^	34.51 ± 7.99^ab^

a, b, and c results refer to average of triplicates ± SD. The mean difference is significant (*p* < 0.05). Same letter in column was used as a notation for statistical analysis; CK (control): *A. thaliana* were cultivated at sterile water.
