# Supplementary material for: Profiles of Bacillus spp. Isolated from the Rhizosphere of Suaeda glauca and Their Potential to Promote Plant Growth and Suppress Fungal Phytopathogens
Source: J Microbiol Biotechnol. 2021 Jul 15;31(9):1231–40. doi: 10.4014/jmb.2105.05010 (PMC9706026; doi:10.4014/jmb.2105.05010)
Supplement: Supplementary file 1 [file jmb-31-9-1231-supple.pdf]

J. Microbiol. Biotechnol.
https://doi.org/10.4014/jmb.2105.05010

Additional File

Supporting information 1

Table S1. The source information of twelve plant-pathogenic fungi.

Plant-pathogenic fungi	Source(Isolation from root or surface)	Store
F. oxysporum	*Citrullus lanatus*	IPEP, BAAFS
F. graminearum Sehw	*Triticum aestivum*	IPEP, BAAFS
R. cerealis van	*Cucumis sativus*	IPEP, BAAFS
G. graminis (sacc.)	*Triticum aestivum*	IPEP, BAAFS
B. cinereal Pers.	*Vitis vinifera*	IPEP, BAAFS
B. dothidea	*Lycopersicon esculentum*	IPEP, BAAFS
C. gloeosporioides Penz	*Camellia sinensis*	IPEP, BAAFS
F. oxysporum f. Sp. niveum	*Citrullus lanatus*	IPEP, BAAFS
C. capsici (syd.) Butl.	*Capsicum annuum*	IPEP, BAAFS
F. verticillioides	*Gossypium* spp.	IPEP, BAAFS
F. oxysporum f. Sp. lilii	*Lilium brownii*	IPEP, BAAFS
F. oxysporum f. Sp. vesinfectum (Atk)	*Citrullus lanatus*	IPEP, BAAFS

Note: IPEP, BAAFS is an abbreviation for Institute of Plant and Environmental Protection of Beijing Academy of Agricultural and Forestry Sciences.

Supporting information 2

Table S2. GenBank accession number and identification analysis of 20 *Bacillus* sp. strains and single *Brevibacterium* sp. strain.

Species	Strain number	Identify (%)	Accession number
B. velezensis	B1	99-100	MK038915
	B2	99-100	MK038916
	B4	99-100	MK038918
	B5	99-100	MK038919
	B7	99-100	MK038921
	B10	99-100	MK038924
	B13	99-100	MK038927
	B21	99-100	MK038935
B. subtilis	B3	100	MK038917
B. pumilus	B6	99-100	MK038920
	B8	99-100	MK038922
	B9	99-100	MK038923
	B14	99-100	MK038928
	B17	99-100	MK038931
B. aryabhattai	B11	99-100	MK038925
	B12	99-100	MK038926
	B15	99-100	MK038929
	B16	99-100	MK038930
	B19	99-100	MK038933
	B20	99-100	MK038934
B. frigoritolerans	B18	98	MK038932

Supporting information 3

Table S3. Antifungal spectrum of the four Bacillus sp. strains against 12 fungal pathogens.

The tested strains	The width of inhibition zone (mm)			
	B1	B5	B16	B21
F. oxysporum	9.78 ± 0.68	9.61 ± 0.75	9.44 ± 0.34	7.17 ± 0.29
F. graminearum Sehw	7.89 ± 0.67	9.33 ± 0.44	10.39 ± 1.62	1.30 ± 0.09
R. cerealis van	8.17 ± 0.17	8.78 ± 0.38	4.00 ± 0.33	14.17 ± 1.04
G. graminis (sacc.)	12.83 ± 0.50	12.72 ± 0.98	10.00 ± 0.44	7.17 ± 0.29
B. cinereal Pers.	11.17 ± 0.73	10.50 ± 0.44	12.89 ± 1.80	10.67 ± 0.29
B. dothidea	8.72 ± 1.00	9.11 ± 0.98	12.74 ± 0.51	13.33 ± 0.76
C. gloeosporioides Penz	10.89 ± 0.79	10.67 ± 0.00	9.72 ± 0.25	7.67 ± 0.76
F. oxysporum f. Sp.niveum	10.33 ± 0.44	9.78 ± 0.19	10.33 ± 1.09	10.67 ± 0.29
C. capsici (syd.) Butl.	12.72 ± 0.42	14.72 ± 0.59	15.33 ± 1.17	13.50 ± 0.50
F. verticillioides	13.61 ± 0.70	10.78 ± 0.59	9.17 ± 0.50	7.17 ± 0.29
F. oxysporum f. Sp. lilii	8.83 ± 0.60	10.33 ± 0.50	11.22 ± 1.25	11.00 ± 0.00
F. oxysporum f. Sp. vesinfectum (Atk)	11.33 ± 1.09	10.00 ± 0.17	12.33 ± 0.17	11.50 ± 0.87
